# PCM-cap to provide thermal comfort for human head

**DOI:** 10.1186/2046-7648-4-S1-A81

**Published:** 2015-09-14

**Authors:** Yigit Can Sezgin, Murat Celik

**Affiliations:** 1 Department of Mechanical Engineering, Bogazici University, Istanbul, Turkey

## Introduction

Motorcycles are one of the most widespread transportation vehicles in the world. However, in hot summer days wearing a motorcycle helmet can be exhaustive and because of high temperatures, the rider may experience loss of concentration and high stress [1]. In order to prevent these effects, phase change materials (PCM)-caps, made from plastic and are filled with two different PCM, are produced and tested. PCM have specific phase change temperatures for different uses. Because a material needs higher heat energy during the phase change, these materials are widely used for thermal energy storage purposes. In this paper, a heat transfer model of the PCM-cap inside the motorcycle helmet is examined. Two different PCM-caps are tested.

## Methods

The heat transfer model of the experiment was accomplished using the finite difference method. It was assumed that the human head is spherical and the model consisted of the following layers: brain, skull, fat, inner skin, outer skin, hair, PCM-cap, insulation and outer shell of the helmet [2]. For the heat transfer model, the bio-heat transfer equation was used and the necessary values taken from other academic studies. It was also assumed that the core of the human head and the outer shell of the helmet have constant temperatures. In the experiment, the temperature of the outer shell of the helmet was kept constant by infrared heater and the temperature between the hair and PCM-cap as measured using K-type thermocouple.

## Results

**Figure 1:**
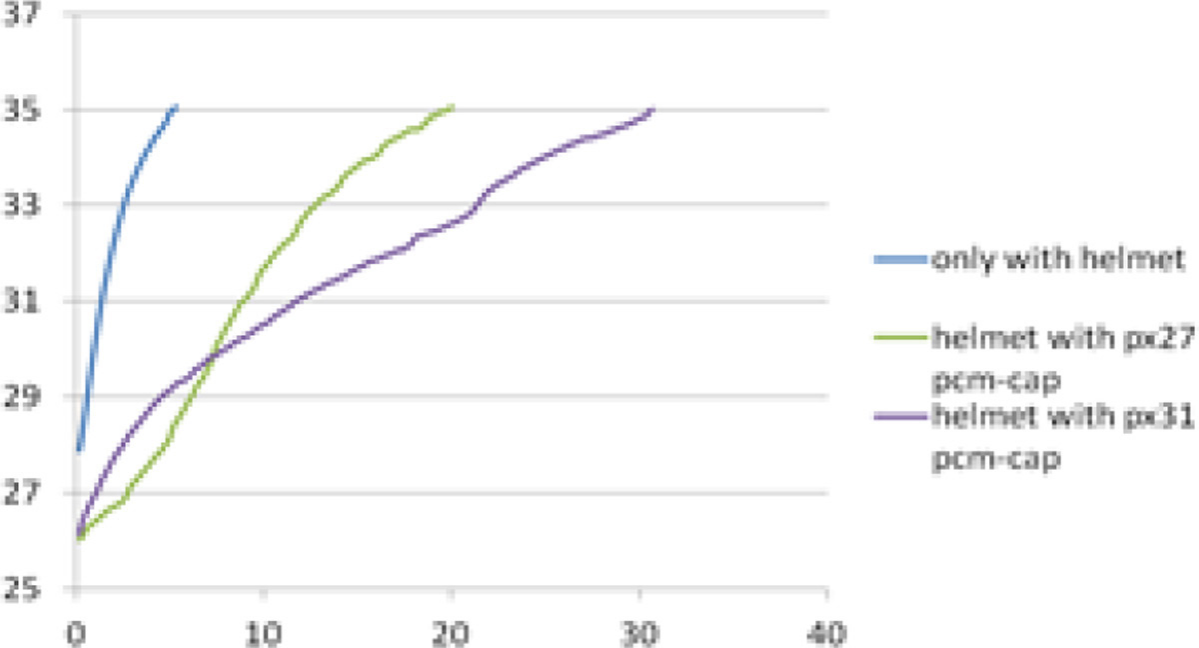


In the experiment, 84.5 grams of PCM was used in each PCM-cap. Two different PCM products, px27 and px31, which are the products of Rubitherm company, were used. A thermocouple was used to measure temperature (°C) as a function of time (in minutes) and is presented in Figure 1.

## Discussion

Using PCM-cap inside the motorcycle helmet increased the time to reach the temperature of 35 °C, which is assumed to be the maximum temperature for thermal comfort of human head [3].

## Conclusion

A PCM-cap inside a motorcycle helmet increases the thermal comfort duration of a motorcycle rider. Therefore, accidents because of the loss of concentration and raised thermal stress may be prevented.
